# Clinical outcomes of a CT protocol for simultaneous examination of the aorta and coronary artery in patients with aortic aneurysm

**DOI:** 10.3389/fcvm.2023.1144444

**Published:** 2023-04-12

**Authors:** Hoyoung Kim, Jihoon Kim, Yeon Hyeon Choe, Sung Mok Kim

**Affiliations:** ^1^ Sungkyunkwan University School of Medicine, Seoul, Republic of Korea; ^2^ Division of Cardiology, Department of Internal Medicine, Samsung Medical Center, Seoul, Republic of Korea; ^3^ Cardiovascular Imaging Center, Heart Vascular Stroke Institute, Samsung Medical Center, Seoul, Republic of Korea; ^4^ Department of Radiology, Samsung Medical Center, Seoul, Republic of Korea

**Keywords:** aortic aneurysm, coronary artery disease, clinical outcomes, multidetector computed tomography, computed tomography angiography

## Abstract

**Objectives:**

In patients with aortic aneurysm (AA), coronary artery disease (CAD) increases the risk of perioperative complications and even asymptomatic CAD is associated with adverse clinical outcomes. We aimed to compare coronary-aorta CT (CACT) with thoracoabdominal CT angiography (Aorta CT) for CAD management and clinical outcomes in these patients.

**Methods:**

We enrolled 479 patients undergoing CACT and 693 patients undergoing Aorta CT as an initial CT scan for AA. The primary outcome was a composite of all-cause death or myocardial infarction (MI) at 3 years after CT. The secondary outcomes were subsequent CAD management and invasive coronary angiography (CAG).

**Results:**

After index CT scan, the CACT group had a significantly higher rate of coronary revascularization compared with the Aorta CT group (10.7% vs. 3.8%, *p* < 0.001) but a lower probability of diagnostic CAG among total invasive CAG (32% vs. 55%, *p* < 0.001). At 3 months after the CT scan, the prescription rates of statins (65.8% vs. 44.6%, *p* < 0.001) and antiplatelet agents (57.6% vs. 43.9%, *p* < 0.001) were higher in the CACT group. During follow-up, the CACT group had a significantly lower incidence of the composite outcome of all-cause death or MI (adjusted HR 1.72, 95% CI 1.07–2.78, *p* = 0.027) than the Aorta CT group.

**Conclusion:**

Among patients with AA, CACT was associated with a higher rate of subsequent CAD management and a lower risk of all-cause death or MI compared to Aorta CT. When evaluating with AA using CT, simultaneous coronary and aortic evaluation using CACT would be recommended over Aorta CT.

## 1. Introduction

Patients with aortic aneurysm (AA) are at increased risk of coronary artery disease (CAD) related to traditional cardiovascular risk factors (e.g., smoking, hypertension, and diabetes mellitus) and common pathways (e.g., atherosclerosis and inflammation) (1, 2). The prevalence of CAD has been reported to be up to 65% in this population (3). In patients with AA, CAD increases the risk of perioperative complications such as myocardial ischemia and death (3–5), and even asymptomatic CAD is associated with adverse clinical outcomes (4, 6). Therefore, screening for CAD is clinically important in patients with AA (4). In previous studies, invasive coronary angiography (CAG) was the main modality used to detect CAD (3, 6). Invasive CAG, however, is accompanied by procedural complications including stroke, vascular injury, and local hematoma (7). As an alternative, coronary computed tomography (CT) angiography is a useful technique for evaluating CAD (8, 9).

Thoracoabdominal CT angiography is a standard method for evaluating AA, but non-electrocardiogram (ECG)-gated thoracoabdominal CT angiography has limitations when assessing and evaluating CAD because of motion artifacts. Although traditional ECG-gated coronary CT angiography has high accuracy for detecting CAD (10, 11), the scan field is limited from the carina to the base of the heart, which limits evaluation of the aortic arch and abdominal aorta. There was a previous attempt to evaluate the coronary artery and aorta simultaneously using ECG-gated 64-channel thoracoabdominal CT (12). Although that study demonstrated the feasibility of ECG-gated thoracoabdominal CT scan for coronary evaluation, the sample size was small with 28 patients, and clinical outcomes were not assessed. With the recent increase in transcatheter aortic valve implantation, the dedicated coronary-aorta CT protocol (CACT), which can evaluate the coronary arteries and aorta simultaneously, has been established for pre-procedural evaluation (13). Therefore, we investigated the relationship between CACT and subsequent CAD management and clinical outcomes compared with conventional thoracoabdominal CT angiography (Aorta CT) in patients with AA.

## 2. Material and methods

### 2.1. Study population

Between January 2010 and May 2021, a total of 8,491 patients underwent CACT or Aorta CT at Samsung Medical Center. The choice of CT protocol was driven by clinician preference. Patients were divided into two groups based on the first CT scan protocols. Of 8,491 patients, 4,868 underwent CACT, and 3,623 underwent Aorta CT. Subjects without AA (1,940 in the CACT group and 2,193 in the Aorta CT group) were excluded. In this study, the presence of AA was defined as an aortic segment exceeding the certain diameter in specific area: ascending aorta ≥40 mm, aortic arch and descending thoracic aorta ≥30 mm, and abdominal aorta ≥20 mm, which were 1.5 times larger than the patient's normal segment in the aorta (14, 15). Subjects with aneurysms at other sites, including the left ventricle or small arteries including the renal, hepatic, and splenic artery, were also excluded (141 in the CACT group and 114 in the Aorta CT group). Furthermore, patients who had history of AA repair (78 in the CACT group and 174 in the Aorta CT group) or who had underlying diseases including genetic vascular disease, vasculitis, congenital disease, valvular heart disease, heart transplantation status, and aneurysms not caused by atherosclerosis (2,230 in the CACT group and 449 in the Aorta CT group) were excluded. Finally, 479 patients with AA who underwent CACT and 693 patients with AA who underwent Aorta CT were included in this study (Supplementary Figure S1).

### 2.2. CT protocol

CT examinations of the coronary artery and aorta were performed using 2nd or 3rd-generation dual-source CT scanners (Somatom Force or Somatom Definition Flash, Siemens Medical Solutions, Forchheim, Germany). Retrospective ECG-gated helical mode with tube current modulation was used for coronary evaluation, followed by prospective ECG-triggered high pitch helical mode for aorta evaluation. The tube voltage and tube current/exposure time product were adjusted according to patient body size as follows: tube voltage, 80–100 kV; tube current/exposure time product, 185–450 mAs; collimation, 2 mm × 192 mm × 0.6 mm or 2 mm × 128 mm × 0.6 mm; gantry rotation time, 250 or 280 ms. A bolus of 50–60 ml of contrast material (Iomeron 400; Bracco, Milan, Italia) was injected into the antecubital vein, followed by 25–30 ml of saline chaser at 4–5 ml/sec for cardiac scan, and a bolus of 60–70 ml of contrast material was injected followed by 40 ml of saline chaser at 4 ml/sec for aorta scan.

CT examinations of the thoracoabdominal aorta were performed using a high-definition CT scanner (Discovery CT 750 HD FREEdom Edition, GE Healthcare, Milwaukee, WI, USA) with a 64 × 0.625 mm detector collimation, Z–coverage 40 mm with an increment of 35 mm and gantry rotation time 350 ms, and field of view of 25 cm. A bolus of 110 to 130 ml of contrast material (Iomeron 300, Bracco; Xeneticx 300, Guerbet, Roissy, France; Omnipaque 300, GE Healthcare, Princeton, NJ, USA; Ultravist 300, Bayer-Schering, Berlin, Germany) was injected into the antecubital vein followed by 40 ml of saline chaser at 3.5–4 ml/sec.

The anatomic coverage for aorta evaluation was the same between the two CT protocols, from the mid clavicle to the symphysis pubis (Supplementary Figure S2).

### 2.3. Clinical data collection

All clinical, laboratory, and image data were collected from medical records. Serum creatinine level was collected before and after CT scans. The estimated glomerular filtration rate was calculated using the Chronic Kidney Disease Epidemiology Collaboration equation (16). The definition of obstructive CAD was based on CT findings showing 50% or greater stenosis. The outcomes were collected by comprehensive reviewing medical records.

### 2.4. Outcomes

The primary outcome was a composite of all-cause death or nonfatal myocardial infarction (MI) during 3 years after the CT scan. MI was defined as elevated cardiac enzyme levels, such as troponin I or myocardial band fraction of creatine kinase, greater than the upper limit of the normal range with either ischemic symptoms or electrocardiography changes indicating ischemia and that required subsequent hospitalization.

The secondary outcomes consisted of impacts of CT protocols on subsequent CAD management and safety of CT scans. First, impact of CT protocols on subsequent CAD management included the proportion of diagnostic only CAG among total invasive CAG procedures, incidence of coronary revascularization (percutaneous coronary revascularization or coronary artery bypass graft) during 3 years of follow-up after CT scan, and the prescription rates of statins or antiplatelet agents within 3 months after CT scan. Diagnostic only CAG was defined as an invasive CAG not followed by revascularization. The general indication of coronary revascularization included left main coronary artery with diameter stenosis ≥50% and major epicardial arteries or large side branches with diameter stenosis ≥70% or 50%–70% with ischemic symptoms or signs. Second, the safety of CT scans was assessed by radiation exposure and contrast-induced nephropathy (CIN). Radiation exposure was calculated as the total dose length product (DLP). CIN was defined as either a 25% increase in baseline serum creatinine or a 0.5 mg/dl increase in absolute serum creatinine value within 72 h after CT scan (17).

### 2.5. Statistical analysis

Continuous variables are presented as mean ± standard deviation, whereas categorical data are presented as frequency or percentage. Shapiro-Wilk test was used to determine whether data were normally distributed. Student's *t*-test was used to determine whether there were significant differences for normally distributed data, whereas the Wilcoxon rank-sum test was used for nonparametric data. Categorical variables were analyzed using Fisher's exact test. Clinical event rates were calculated by Kaplan-Meier censoring estimates and presented with the cumulative incidence. Log-rank tests were used to compare survival curves between CACT and Aorta CT groups. Cox proportional hazard regression was used to calculate hazard ratio (HR) and 95% confidence interval (CI) with adjustments for age, sex, hypertension, diabetes mellitus, medication (statins and antiplatelet agents) before CT scan, and location of the aneurysm.

A sensitivity analysis comparing the primary outcome between the CACT group and the Aorta CT group was conducted, excluding patients who underwent AA repair during follow-up. Subgroup analyses were conducted according to age, sex, diabetes mellitus, medication history before CT scan, and size and location of AA. All probability values are two-sided and statistical significance was defined as *p*-value <0.05. SPSS version 27 and R version 4.2.0 (R Foundation for Statistical Computing) were used for all statistical analyses.

## 3. Results

Table 1 shows the baseline characteristics of 479 patients who underwent CACT and 693 patients who underwent Aorta CT. The CACT group was younger (69.9 ± 10.1 vs. 71.3 ± 10.0 years, *p* = 0.022) and had higher body mass index (24.5 ± 3.3 vs. 24.0 ± 3.2 kg/m^2^, *p* = 0.011) and lower prevalence of abdominal AA (54.1% vs. 71.4%, *p* < 0.001), but had higher prevalence of hypertension (75.2% vs. 65.2%, *p* < 0.001) and dyslipidaemia (64.1% vs. 46.3%, *p* < 0.001) compared with the Aorta CT group.

**Table 1:** Baseline characteristics of the patients who underwent CACT or aorta CT.

	CACT (*n* = 479)	Aorta CT (*n* = 693)	*p* value
Age (years)	69.9 ± 10.1	71.3 ± 10.0	0.022
Sex (male, %)	344 (71.8%)	523 (75.5%)	0.161
Height (cm)	163.9 ± 9.1	164.1 ± 9.2	0.747
Weight (kg)	66.3 ± 11.8	65.1 ± 11.3	0.071
BMI (kg/m^2^)	24.5 ± 3.3	24.0 ± 3.2	0.011
BSA (m^2^)	1.73 ± 0.2	1.71 ± 0.2	0.200
Hypertension (%)	360 (75.2%)	452 (65.2%)	<0.001
Diabetes mellitus (%)	105 (21.9%)	142 (20.5%)	0.555
Dyslipidemia (%)	307 (64.1%)	321 (46.3%)	<0.001
Renal dysfunction (%)^a^	99 (20.7%)	171 (24.7%)	0.121
Location of aneurysm (AAA)	259 (54.1%)	498 (71.4%)	<0.001
Statins	102 (21.3%)	112 (16.2%)	0.025
Antiplatelet agents	79 (16.5%)	116 (16.7%)	0.911
History of coronary revascularization	69 (14.4%)	111 (16.0%)	0.452

Continuous variables are presented as mean ± standard deviation. Categorical data are presented as frequency and percentage. AAA, abdominal aortic aneurysm; Aorta CT, thoracoabdominal aorta computed tomography angiography protocol; BMI, body mass index; BSA, body surface area; CACT, coronary-aorta computed tomography protocol; eGFR, estimated glomerulus filtration rate.

^a^ Renal dysfunction was defined as eGFR <60 ml/min/1.73 m^2^.

Obstructive CAD was detected in 215 of 479 patients (44.9%) in the CACT group. During the follow-up period, 90 patients in the CACT group and 156 in the Aorta CT group underwent open repair of AA (18.8% vs. 22.5%, *p* = 0.143). Among 246 patients who underwent AA open repair, 3 patients developed MI within 30 days of surgery. Two out of 3 patients with MI underwent invasive CAG and had a significant stenosis of 90% or more in major epicardial coronary arteries. All 3 cases were in the Aorta CT group.

### 3.1. Subsequent CAD management after CT scan

**Figure 1:**
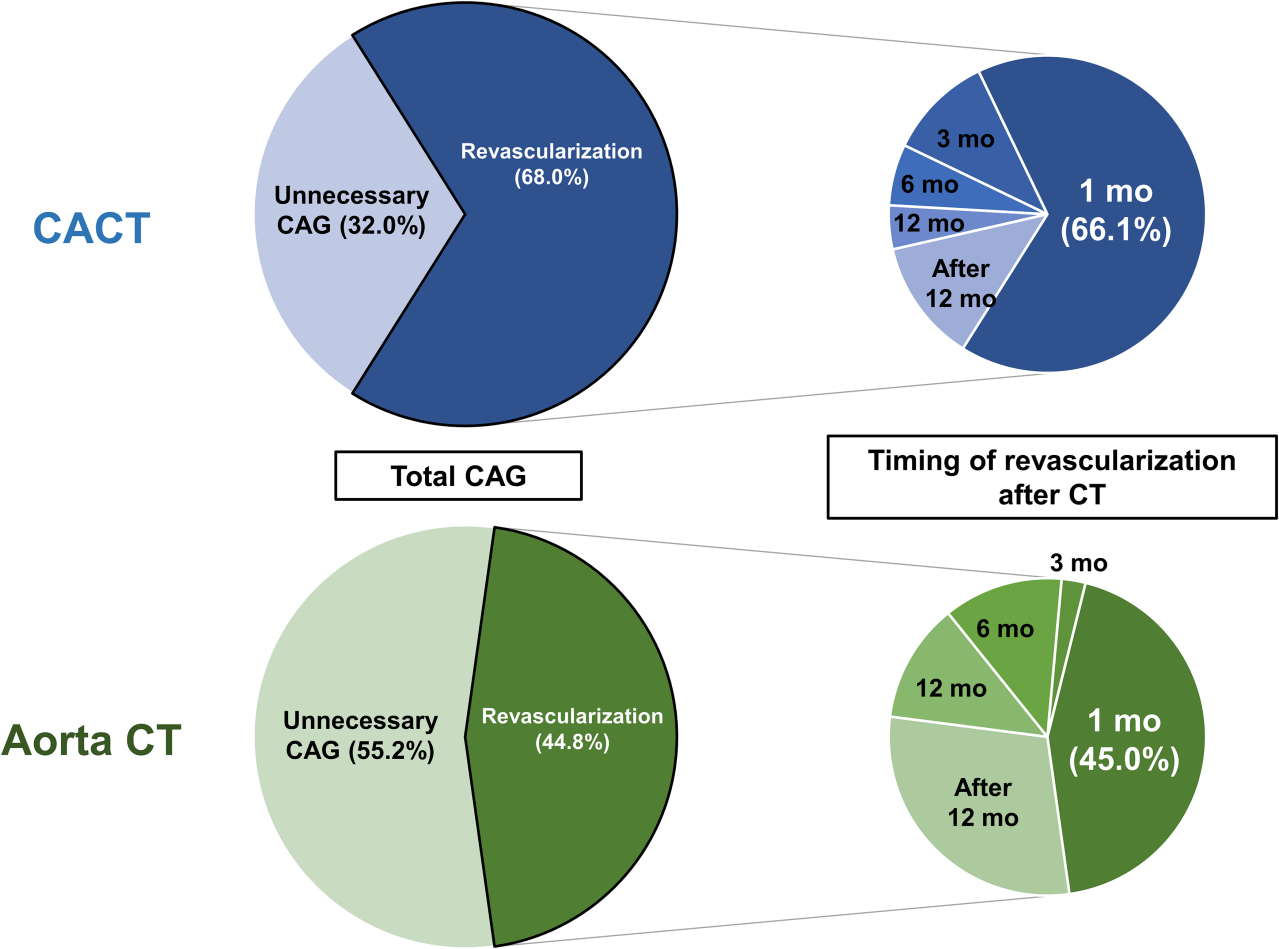
Patterns of invasive CAG and timing of coronary revascularization after CT scan. **Left** panels describe the proportions of diagnostic only CAG and coronary revascularization among total invasive CAG after CT scan. **Right** panels describe the timing of coronary revascularization after CT scan. Aorta CT, thoracoabdominal aorta CT angiography protocol; CACT, coronary-aorta CT protocol; CAG, coronary angiography; CT, computed tomography; mo, month.

There were 132 invasive CAGs during the 3 years after the index CT scan. The proportion of diagnostic only CAG among total invasive CAG procedures was significantly lower in the CACT group than the Aorta CT group (32.0% vs. 55.2%, *p* < 0.001) (left panel in Figure 1). During the 3-year follow-up, the CACT group had a significantly higher incidence of coronary revascularization compared with the Aorta CT group (11.2% vs. 4.0%, *p* < 0.001) (Supplementary Figure S3). Most revascularizations were performed within 1 month after CT scan in the CACT group, but not in the Aorta CT group (66.1% vs. 45.0%, *p* < 0.001) (right panel in Figure 1).

**Figure 2:**
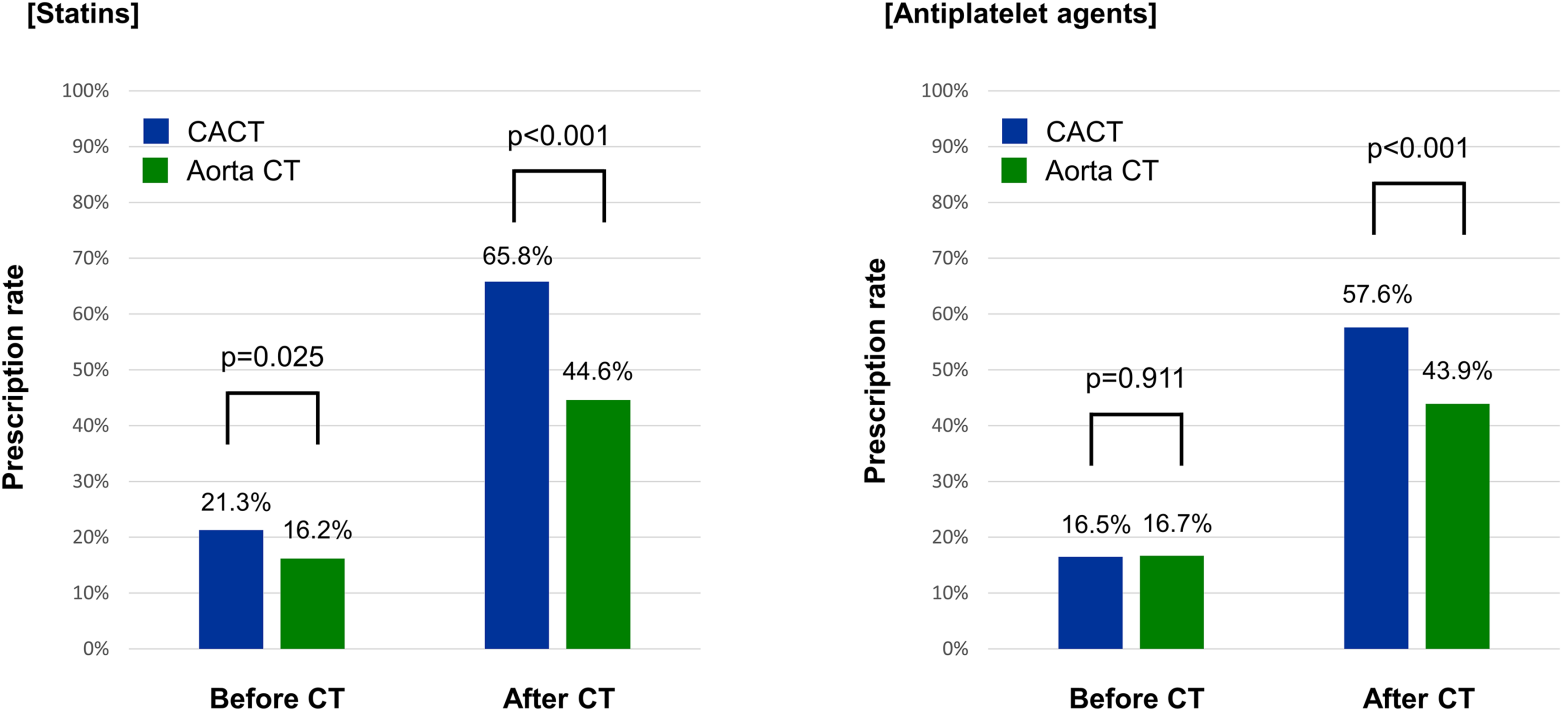
Prescription rates of cardiovascular medications before and after CT scan. Aorta CT, thoracoabdominal aorta CT angiography protocol; CACT, coronary-aorta CT protocol; CT, computed tomography.

In addition, the CACT group had a significantly higher prescription rate of statins (65.8% vs. 44.6%, *p* < 0.001) and antiplatelet agents (57.6% vs. 43.9%, *p* < 0.001) than the Aorta CT group at 3 months after CT scan (Figure 2).

### 3.2. Clinical outcomes according to CT protocol

**Figure 3:**
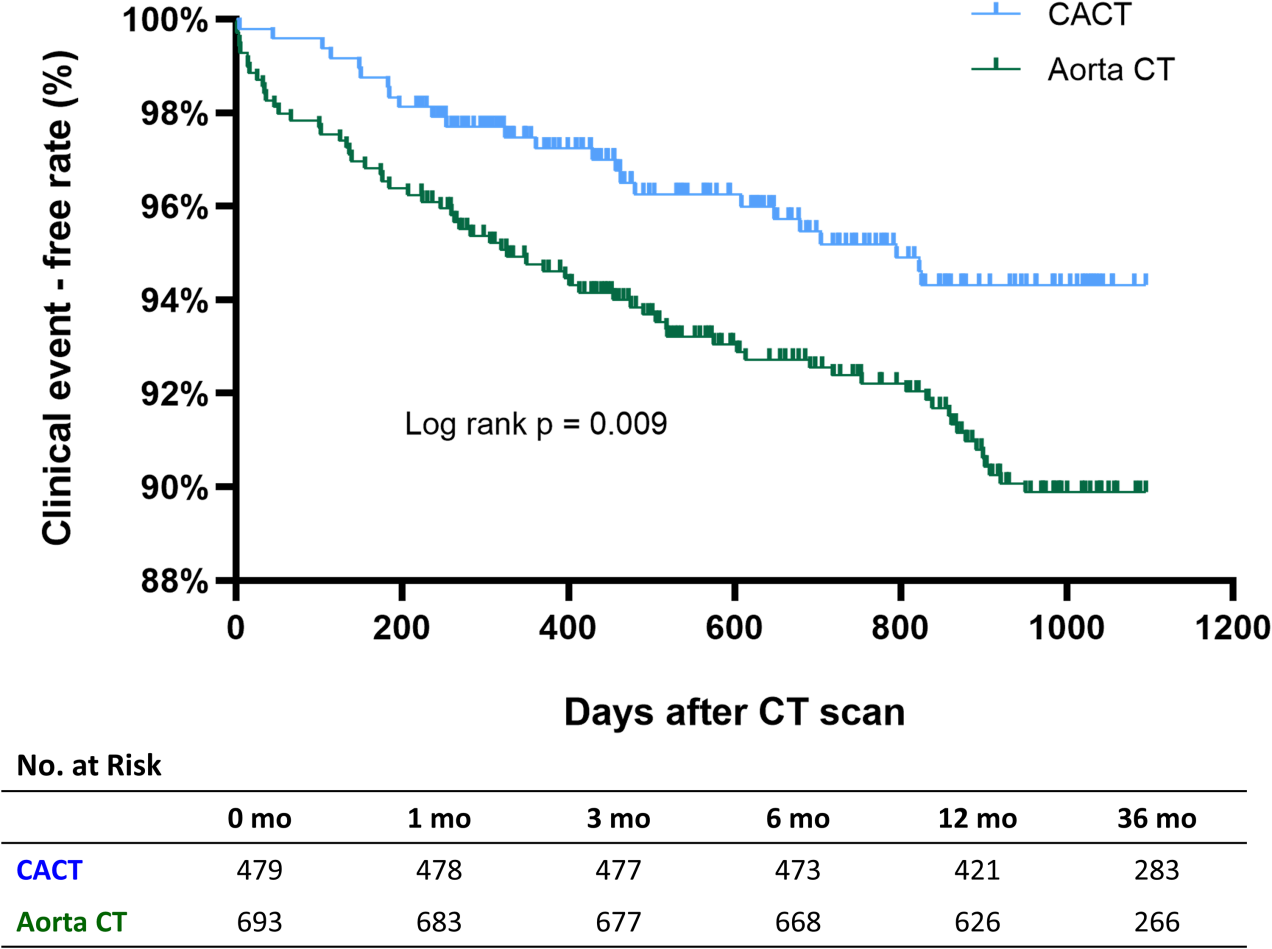
Incidence of all-cause death or myocardial infarction according to CT protocol. Aorta CT, thoracoabdominal aorta CT angiography protocol; CACT, coronary-aorta CT protocol; CT, computed tomography.

There were 76 deaths and 14 nonfatal MIs (23 deaths and 2 MIs in the CACT group, and 53 deaths and 12 MIs in the Aorta CT group) during the 3 years of follow-up. The incidence of death or nonfatal MI was significantly lower in the CACT group compared with the Aorta CT group (5.7% vs. 9.5%, adjusted HR 1.71, 95% CI 1.06–2.76, *p* = 0.028) (Figure 3).

### 3.3. Independent predictors of death or nonfatal MI

In multivariable analysis, age, sex, renal dysfunction, and CT protocol were independent predictors of death or nonfatal MI at 3 years (Table 2). Multivariable Cox regression analysis including the medications after CT is presented in Supplementary Table S1.

**Table 2:** Independent predictors for composite of all-cause death or myocardial infarction in patients with aortic aneurysm.

	Hazard ratio (95% CI)	*p* value
Aorta CT (vs. CACT)	1.71 (1.06–2.76)	0.028
Age	1.03 (1.01–1.06)	0.010
Sex	2.51 (1.34–4.72)	0.004
Obesity (BMI ≥ 30 kg/m^2^)	0.43 (0.06–3.12)	0.403
Hypertension	1.36 (0.81–2.29)	0.239
Diabetes mellitus	1.29 (0.80–2.08)	0.291
Statin	1.04 (0.53–2.04)	0.916
Antiplatelet agent	1.24 (0.63–2.44)	0.535
Renal dysfunction^a^	2.07 (1.33–3.22)	0.001
History of coronary revascularization	0.60 (0.33–1.11)	0.104
Location of aortic aneurysm	1.10 (0.66–1.83)	0.725

This table shows the results of cox proportional hazard regression. Aorta CT, thoracoabdominal aorta computed tomography angiography protocol; BMI, body mass index; CACT, coronary-aorta computed tomography protocol; CI, confidence interval; eGFR, estimated glomerulus filtration rate.

^a^ Renal dysfunction was defined as eGFR <60 ml/min/1.73m ^2^.

### 3.4. Subgroup analysis

**Figure 4:**
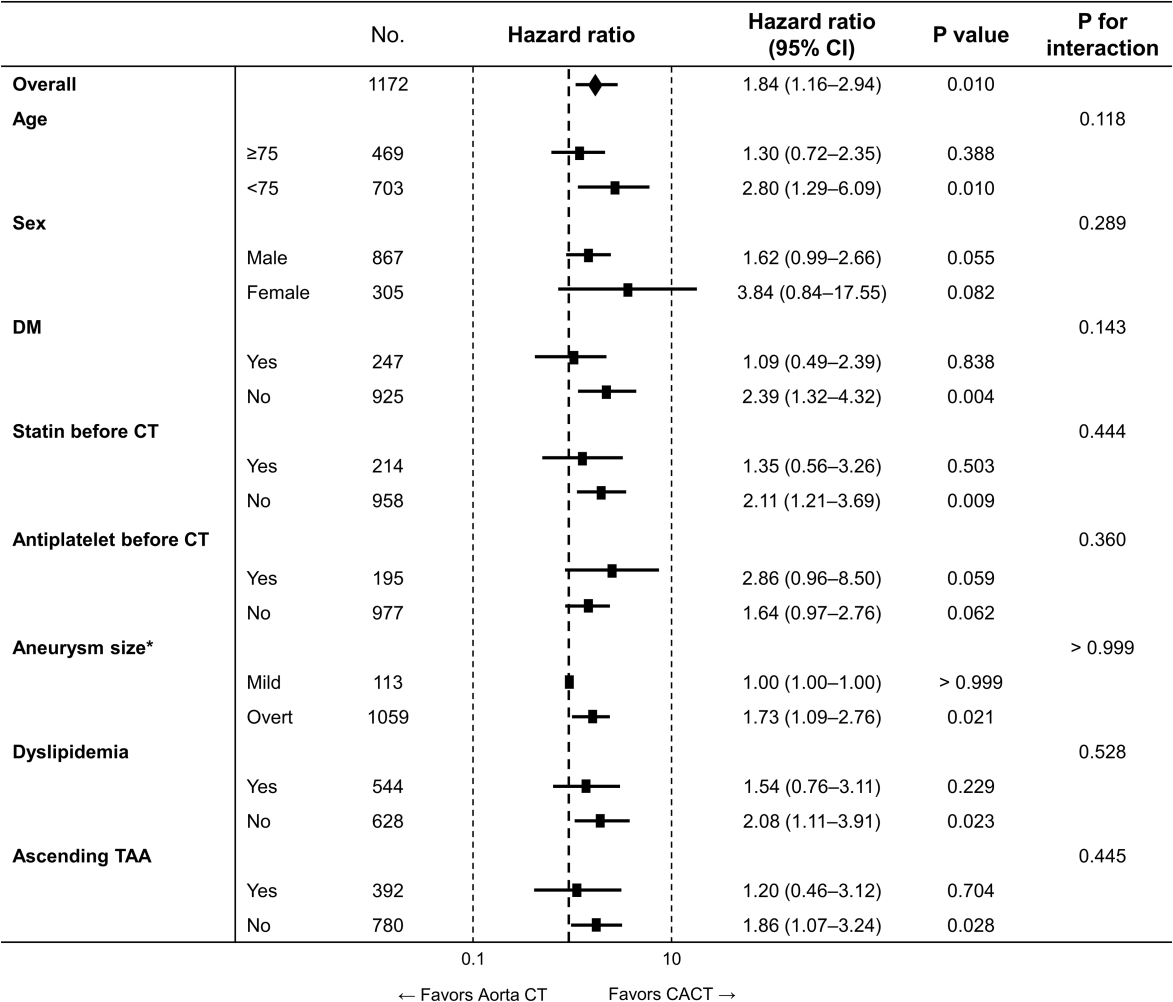
Subgroup analysis for the primary outcome. Aorta CT, thoracoabdominal aorta CT angiography protocol; CACT, coronary-aorta CT protocol; CI, confidence interval; CT, computed tomography; No., number; TAA, thoracoabdominal aortic aneurysm. * Mild form of aneurysm was defined as abdominal aorta with a size between ≥20 and <30 mm or aortic arch or descending thoracic aorta with a size between ≥30 and <35 mm, while overt aneurysm was defined as ascending aorta with a size ≥40 mm, aortic arch or descending aorta with a size ≥35 mm, and abdominal aorta with a size ≥30 mm.

The lower risk of the primary outcome in patients evaluated with CACT was consistent across subgroups (Figure 4).

### 3.5. Radiation exposure and CIN

The mean total DLPs were 611.6 ± 301.4 and 578.8 ± 286.8 mGy·cm in the CACT and the Aorta CT group, respectively. There were no significant differences in radiation dose (611.6 vs. 578.8 mGy·cm, *p* = 0.061) or incidence of CIN (3.6% vs. 2.6%, *p* = 0.347) between the CACT and Aorta CT groups.

### 3.6. Clinical outcome in patients without AA repair

Among patients who did not undergo AA repair during follow-up, the 3-year incidence of death or nonfatal MI was significantly lower in the CACT group compared with the Aorta CT group (5.7% vs. 10.6%, adjusted HR 1.72, 95% CI 1.04–2.84, *p* = 0.035) (Supplementary Figure S4).

## 4. Discussion

In the present study we compared two CT protocols, CACT and Aorta CT, to investigate the relationship between CT protocols and cardiovascular outcomes in patients with AA. The main findings were as follows. First, patients with CACT had a higher incidence of coronary revascularization and higher prescription rate of cardioprotective medications after CT than those evaluated with Aorta CT, while the probability of undergoing unnecessary CAG without revascularization was lower in those evaluated with CACT. Second, patients evaluated with CACT had a significantly lower risk of all-cause death or nonfatal MI at 3 years compared to those evaluated with Aorta CT. Third, there were no significant differences in radiation dose or CIN between the CT protocols.

Asymptomatic CAD is highly prevalent in patients with AA (4, 18). It would be clinically demanding to screen for CAD irrespective of symptoms because a significant proportion of asymptomatic CAD, as high as 51% in a previous report by Fabio et al., meet indications for coronary revascularization (18). Previous studies showed that ECG-gated coronary CT angiography is a feasible alternative to invasive CAG for detection of CAD (19). In our study, patients evaluated with CACT underwent coronary revascularization more frequently than those evaluated with Aorta CT. Most of the coronary revascularizations in patients with CACT were performed within initial 1 month after the CT scan, which suggests that the decision to treat CAD was probably based on the CT findings. Moreover, among patients undergoing invasive CAG, the proportion of diagnostic only CAG that was not followed by revascularization was 32% in patients with CACT but 55% in those with Aorta CT. This finding may be due to the high negative predictive value of coronary CT angiography, which can prevent unnecessary CAG in asymptomatic but high-risk patients such as our study population (20). Therefore, simultaneous evaluation of coronary arteries during aortic CT scans using CACT may aid in effective screening for CAD and subsequent decisions to perform invasive CAG considering coronary revascularization in patients AA. Whether PCI can reduce mortality or MI in patients with stable CAD remains controversial (21). In patients with AA, however, several reports have suggested that appropriate management including coronary revascularization may improve clinical outcomes (4, 22). Functional tests such as stress echocardiography or single-photon emission computed tomography could be alternatives to CACT for the detection of coronary artery disease. However, CACT allows for the simultaneous screening of coronary artery disease with a single test in patients who are planned for CT evaluation for other causes, particularly in those with high-risk factors for coronary artery disease such as aortic aneurysm.

Given that CAD is the leading cause of death in patients with AA (23), coronary evaluation and subsequent management might have affected clinical outcomes in the present study. There were significant differences in medical management between the two CT protocols. After CT scans, both statins and antiplatelet agents were more frequently prescribed in patients with CACT. Underlying disease, such as hypertension and dyslipidemia, might affect the use of cardiovascular medications, but the difference in statin prescription rate between the two CT protocols increased from 5.1% before CT scan to 21.2% after the CT. Pharmacologic therapy is a key management tool for CAD as well as atherosclerotic disease to reduce adverse cardiovascular events. Hence, the guidelines for secondary prevention and risk reduction for atherosclerotic disease recommend statins and antiplatelet agents in patients with coronary artery disease (24). Coronary CT angiography-based strategies are generally associated with increased likelihood of initiation of aspirin and statin in patients with suspected CAD (25). The Scottish Computed Tomography of the Heart (SCOT-HEART) study, a randomized trial comparing coronary CT angiography with standard care alone in patients with suspected CAD, demonstrated that patients with coronary CT angiography had a lower risk of death from coronary heart disease or nonfatal MI (26). In that trial, patients with coronary CT angiography were more likely to have preventive medical therapy than those with standard care alone, which explained the difference in clinical outcomes between the two strategies. In our study, CACT would detect CAD and then guide clinicians to prescribe preventive medications even for cases of subclinical CAD not requiring further invasive CAG or revascularization. Although the relationships between antiplatelet agents and cardiovascular outcomes in patients with CAD remain controversial, a high-risk population such as those with AA are likely to benefit from antiplatelet agents (27). As antiplatelet agents are usually prescribed for patients at high risk of atherosclerotic disease (e.g., stroke) or those undergoing coronary revascularization, the beneficial effect of antiplatelet agents on cardiovascular outcomes may be diluted in our study.

Although coronary artery and aorta examinations are performed simultaneously in CACT scan, there were no significant differences in radiation dose between the CT protocols. The radiation dose of CACT was 611.6 ± 301.4 mGy·cm, which was lower than the 982.5 mGy·cm in a previous study using coronary artery scans with ECG-gated thoracoabdominal 64-detector-row CT angiography (28). Our CACT protocol used high-pitch helical mode for aorta evaluation and lower tube potential for both coronary and aorta scans to reduce radiation exposure (29). It is recommended to use smaller amounts of iodine contrast because high volumes of contrast medium increase risk of CIN (30). In our study, the amount of contrast medium could not be directly compared between CT protocols because the dose was not recorded at the time of each examination of Aorta CT. Between the two CT protocols, however, there was no significant difference in the rate of CIN, a meaningful surrogate marker of overuse of contrast medium.

There are several limitations in the present study. First, this was a retrospective observational study. The choice of CT protocol was at the clinician's discretion. Although the differences in clinical outcomes between the two protocols were significant after adjustment of baseline characteristics, there might have been selection bias. Second, because this was a single-center study, the generalizability of our findings might be limited. For example, the baseline prescription rate of statin was relatively low compared with previous studies including patients with AA (31, 32). After index CT scans, however, the prescription rate of cardiovascular medicine increased to a level similar to that of previous studies, with diagnosis of AAs by CT scans. In addition, the prevalence of obstructive CAD in the CACT group of our study (44.9%) was similar to a previous report (51%) (18). Third, AA repair surgery may have influenced the incidence of death or nonfatal MI. However, there was no death related with surgery, and all 3 cases of perioperative MI with critical coronary artery stenoses occurred in the Aorta CT group. This finding may explain the higher incidence of death or nonfatal MI in patients with Aorta CT that cannot lead to intensified treatment even in the presence of severe CAD. In addition, the higher incidence of death or nonfatal MI with Aorta CT was consistent after excluding patients undergoing AA repair. Fourth, although smoking history is one of the important factors for disease progression, there were missing values (in 29.1% of patients) in smoking status in our study because this was a retrospective study. However, without missing values, there were no significant differences in smoking history between two groups (current or ex-smoker, 40.2% in the CACT vs. 45.8% in the Aorta CT, *p* = 0.132). Last, the sizes and progression rates of AAs, which are prognostic factors in patients with AA, were not available. However, the rate of open repair during follow-up period was not significantly different between patients evaluated with the two CT protocols.

## 5. Conclusion

Among patients with AA, CACT was associated with a higher rate of subsequent CAD management and a significantly lower risk of all-cause death or MI with CACT compared to Aorta CT. Therefore, when evaluating with AA using CT, simultaneous coronary and aortic evaluation using CACT would be recommended over Aorta CT.

## Data Availability

The original contributions presented in the study are included in the article/**Supplementary Material**, further inquiries can be directed to the corresponding authors.
