# Supplementary material for: Clinical outcomes of a CT protocol for simultaneous examination of the aorta and coronary artery in patients with aortic aneurysm
Source: Front Cardiovasc Med. 2023 Apr 12;10:1144444. doi: 10.3389/fcvm.2023.1144444 (PMC10130567; doi:10.3389/fcvm.2023.1144444)
Supplement: Supplementary file 1 [file Datasheet1.docx]

**Supplementary Table 1. Independent predictors of all-cause death or myocardial infarction, including therapies after CT scan in patients with aortic aneurysm.**

|  | Hazard ratio (95% CI) | P value |
| --- | --- | --- |
| Age | 1.04 (1.01–1.06) | 0.007 |
| Sex | 2.50 (1.33–4.69) | 0.004 |
| Obesity (BMI≥30 kg/m2) | 0.41 (0.06–2.96) | 0.375 |
| Hypertension | 1.10 (0.65–1.86) | 0.718 |
| Diabetes mellitus | 1.23 (0.77–1.97) | 0.385 |
| Statin after CT | 0.57 (0.34–0.95) | 0.032 |
| Antiplatelet agent after CT | 1.24 (0.74–2.09) | 0.416 |
| Renal dysfunction* | 2.27 (1.46–3.53) | < 0.001 |
| Location of aortic aneurysm | 1.11 (0.70–1.76) | 0.666 |

**This table shows the results of multivariable cox proportional hazard regression.**

*** Renal dysfunction was defined as eGFR <60 mL/min/1.73m^2^.**

**Abbreviations: BMI, body mass index; CT, computed tomography; CI, confidence interval; eGFR, estimated glomerulus filtration rate.**

**Supplement Figure 1. Flow chart of patient enrolment**


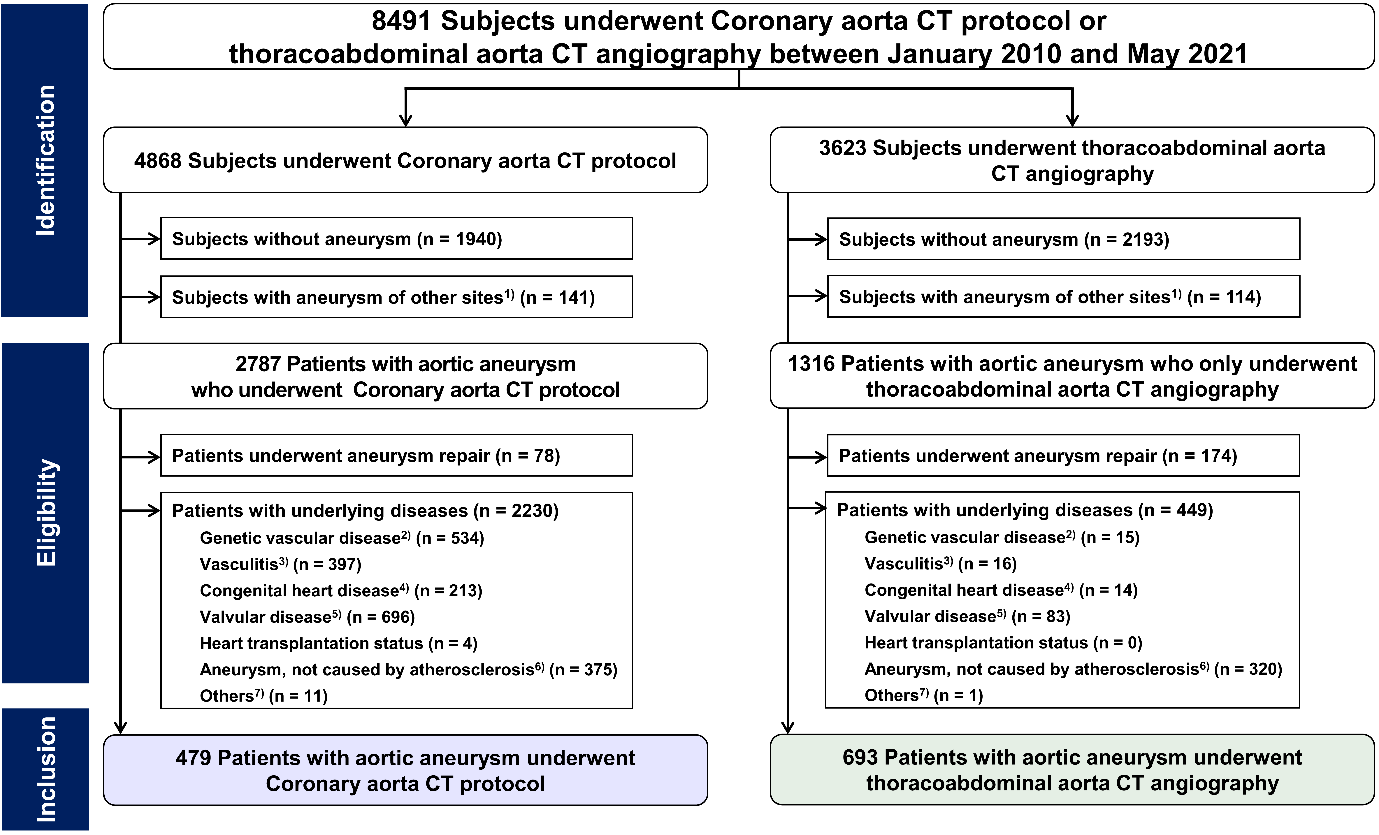


Note: 1) Organs other than left ventricle or small arteries including renal artery, hepatic artery, splenic artery etc; 2) Marfan syndrome, Loeys-Dietz syndrome, Ehler-Dalnos syndrome, familial arterial dissection, and aneurysms; 3) Takayasu’s arteritis, Behcet’s disease, ankylosing spondylitis, giant cell aortitis, fibrosclerosing peri-aortitis, IgG4-related aortitis, Kawasaki disease, polyarteritis nodosa, polymyalgia rheumatica, sarcoidosis; 4) Coarctation of aorta, bicuspid aortic valve, others; 5) Mitral regurgitation, aortic regurgitation, aortic stenosis, mitral stenosis; 6) Mycotic aneurysm, aneurysm with acute aortic syndromes (aortic dissection, acute intramural hematoma, penetrating atherosclerotic ulcer), pseudoaneurysm, ruptured aneurysm, saccular aneurysm; 7) Fibromuscular dysplasia, thromboangitis obliterans, hereditary haemorrhagic telangiectasia.

Abbreviations: CT, computed tomography.

**Supplement Figure 2. Thoracoabdominal CT angiography and coronary aorta CT protocol**


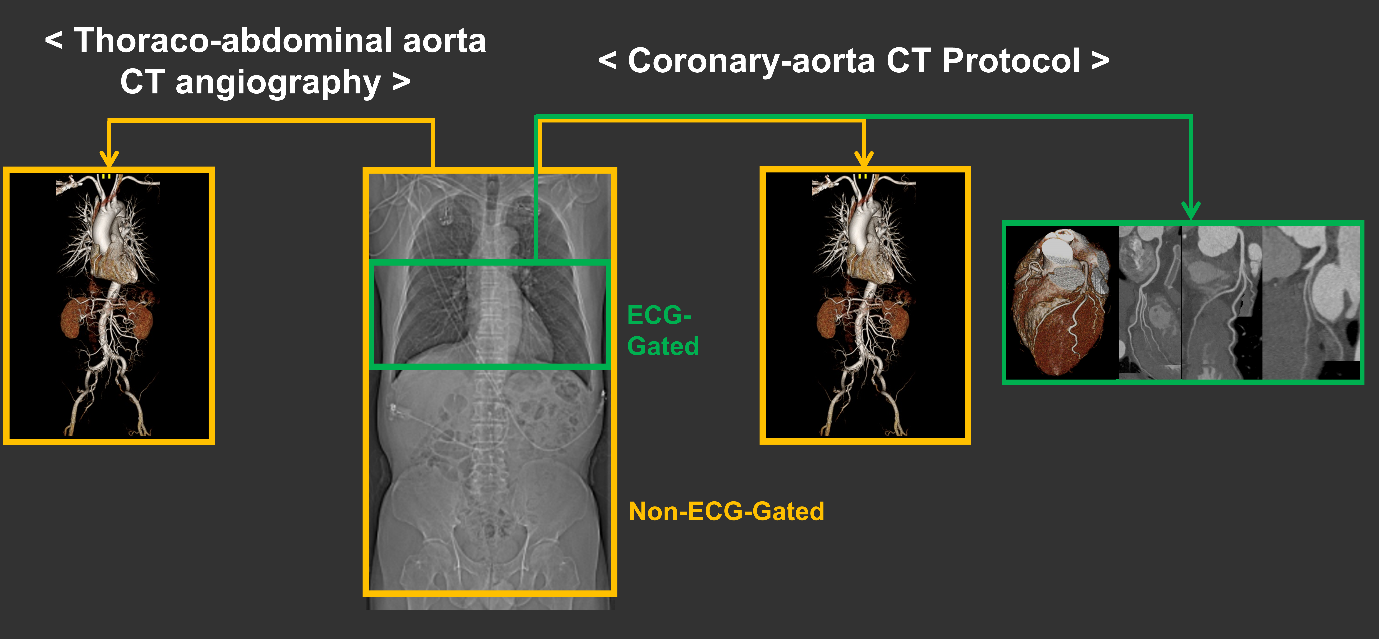


Compared with thoracoabdominal CT angiography, coronary aorta CT angiography provides ECG-gated scan of coronary arteries.

Abbreviations: CT, computed tomography; ECG, electrocardiogram.

**Supplement Figure 3. Incidence of coronary revascularization during the follow-up period after CT scan**

**
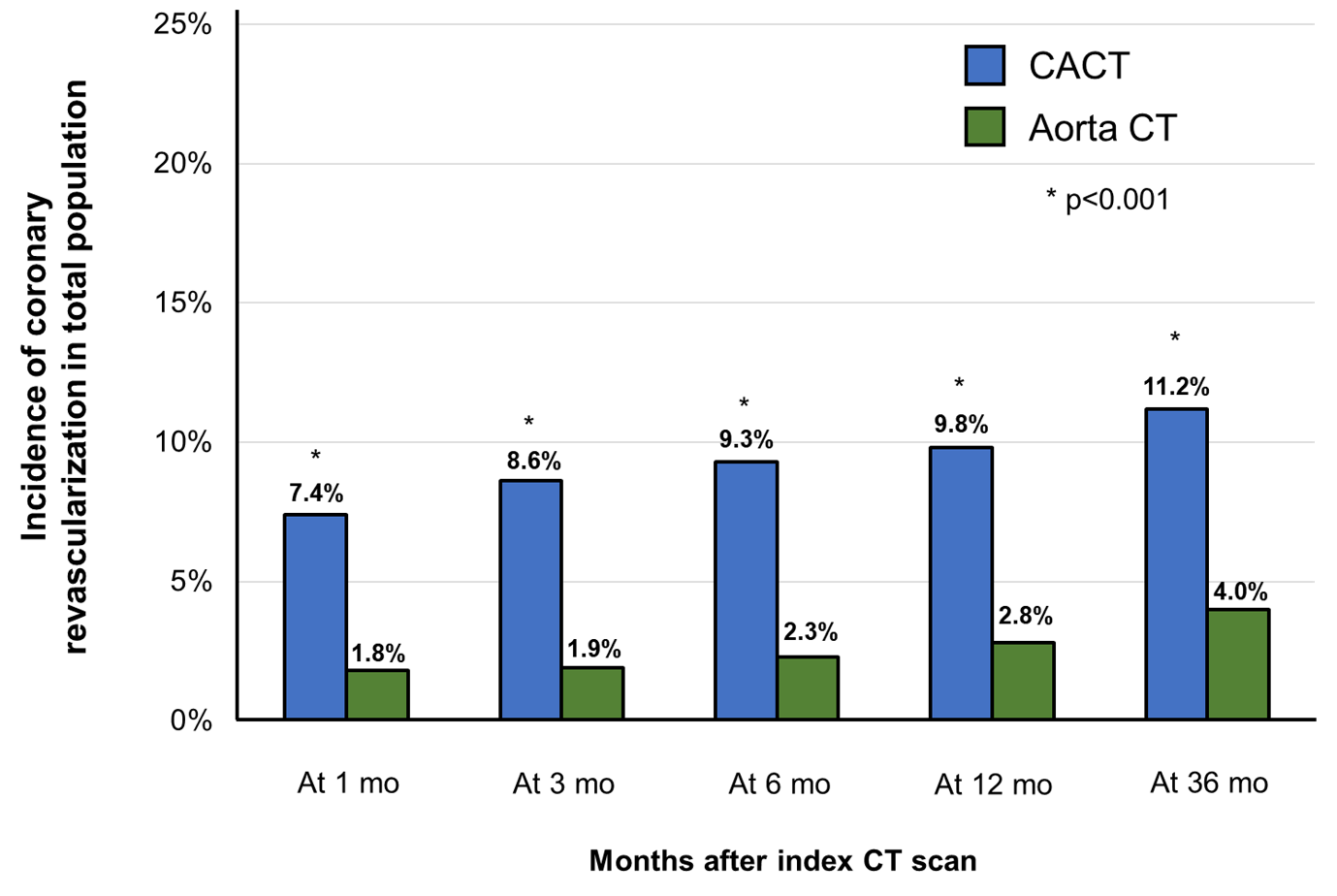
**

* Denotes p value less than 0.001.

Abbreviations: Aorta CT, thoracoabdominal aorta CT angiography protocol; CACT, coronary-aorta CT protocol; CT, computed tomography; mo, month.

**Supplement Figure 4. The incidence of death or nonfatal myocardial infarction at 3 years after CT scan in patients who did not undergo aortic aneurysm repair during follow-up period**


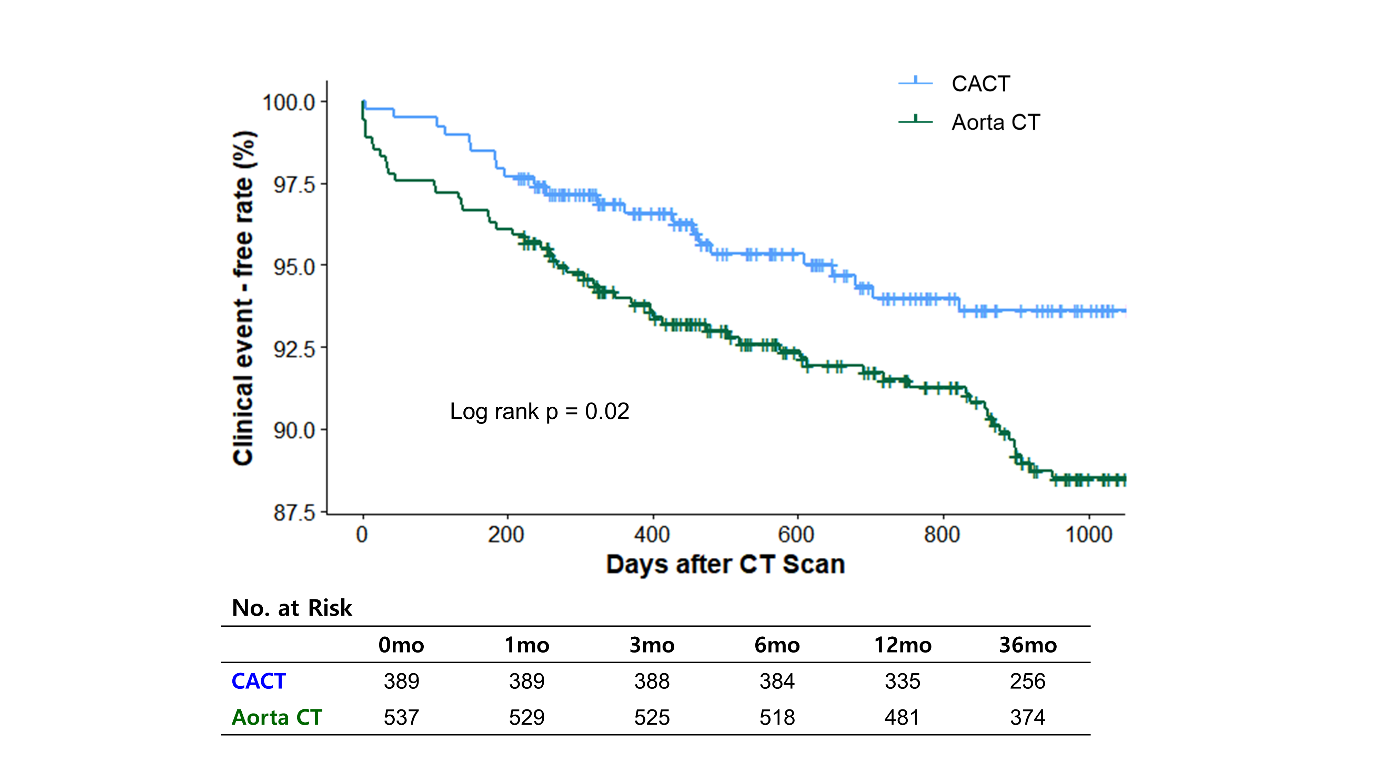


Abbreviations: CACT, coronary aorta computed tomography; CT, computed tomography.
